# Peer supported Open Dialogue in the National Health Service: implementing and evaluating a new approach to Mental Health Care

**DOI:** 10.1186/s12888-022-03731-7

**Published:** 2022-02-22

**Authors:** Catherine Kinane, James Osborne, Yasmin Ishaq, Marcus Colman, Douglas MacInnes

**Affiliations:** 1 Combat Stress, Tyrwhitt House, Oaklawn Road, Leatherhead, KT22 0BX UK; 2 Farm Villa, Kent and Medway NHS and Social Care Partnership Trust, Hermitage Lane, Kent, ME16 9PH UK; 3 Faculty of Medicine, Health and Social Care, Canterbury Christ Church University, North Holmes Road, Canterbury, Kent, CT1 1QU UK

**Keywords:** Open dialogue; mental health; community psychiatry; peer support, Social network, Carer support, Wellbeing, Social adjustment, Functioning

## Abstract

**Background:**

Open Dialogue is an internationally developing approach to mental health care based on collaboration between an individual and their family and social network. Our quest for better approaches to Mental Health Care with improved carer and service user experience led us to develop and test a model of Peer Supported Open Dialogue (POD). There is no research currently looking at the implementation and effectiveness of a standalone POD team in the NHS so we evaluate its implementation, clinical outcomes and value to service users and their families.

**Method:**

A before-after design was used. 50 service users treated by the POD Team were recruited and participants from their family and wider social network. Service user self report questionnaires covering wellbeing, functioning, satisfaction were collected and one carer self report measure; at baseline, three and six months. A clinician reported measure was collected at baseline and six months. Clinicians perceptions of practice were collected following network meetings.

**Results:**

50 service users treated were recruited with a mean age of 35 years with slightly more males than females. Service users reported signficant improvements in wellbeing and functioning. There was a marked increase in perceived support by carers. Over half the meetings were attended by carers. The Community Mental Health Survey showed high satisfaction rates for service users including carer involvement.

**Conclusions:**

The study indicated it was possible to transform to deliver a clinically effective POD service in the NHS. This innovative approach provided continuity of care within the social network, with improved carer support and significant improvements in clinical outcomes and their experiences.

**Trial registration:**

(isrctn.com/ISRCTN36004039. Retrospectively registered 04/01/2019.

**Supplementary Information:**

The online version contains supplementary material available at 10.1186/s12888-022-03731-7.

## Background

Open Dialogue is an approach to working with mental health crises that originated in Western Lapland, Finland [1, 2]. It has gained substantial international interest due to its emphasis on social network support, generating dialogue about the mental health crisis and involving the service user in all decisions regarding treatment [3]. Open Dialogue not only describes a way of being with the other, without conditions, but also a way of organising a mental health service to make dialogue and continuity of care possible. The seven organising principles that emerged from the work in Western Lapland [4, 5] (Ziedonis D, Olson M, Seikkula J: 10 Organizational criteria of open dialogue, unpublished) are:

i. immediate help
ii. a social networks perspective,
iii. flexibility and mobility,
iv. responsibility
v. psychological continuity,
vi. tolerance of uncertainty
vii. dialogism.

POD is empowering of the service user and embraces a social network approach which brings together the social and professional network. This creates a space where service users and their network find words for their experiences. The aim of dialogic practice in Open Dialogue is to actively listen and respond, generating dialogue between all participants. This is unlike traditional treatments where methods or interventions are planned for a specific diagnosis to reduce symptoms or change thinking. In network meetings, clinicians reflect between themselves in the presence of the service user and their network, usually family members, with the aim of making sense of the crisis. Treatment decisions are made by all participants with the expressed aim of avoiding hasty treatment planning. POD is integrative and inherently democratic with transparent decision making.

### Open Dialogue model

Evidence from the Open Dialogue (OD) service in Finland indicated those receiving the service had lower levels of antipsychotic medication use, lower rates of relapse and hospitalisation and were more likely to return to work or education compared with users of traditional services [1, 6]. These positive findings were maintained at a twenty year follow up [7]. Several other countries have embraced the OD approach, with initiatives in the United States and several countries across Europe, including the United Kingdom, Austria, Italy, Germany, Poland, Finland, Norway, Denmark and Australia. Although the evidence to support the application of Open Dialogue looks promising a systematic review of the published evidence for OD interventions [8] reported the studies undertaken were mainly qualitative, cross-sectional and small scale, with a lack of high-quality empirical publications. Issues noted included variable adherence to the model and differences in inclusion criteria leading to low internal validity of the evidence along with potential bias from the researchers. These methodological limitations led the reviewers to question the validity of the conclusions drawn and conclude that more robust research designs were required to assess the effectiveness of Open Dialogue. The review also noted that, of the six quantitative studies included, four were conducted by the developers of open dialogue who also provided the study participant ratings. The other two studies were open trials; one examined changes in only one outcome variable (suicidal ideation) and the other examined a range of outcomes among 16 participants over one year period. Six qualitative studies focused on the personal experiences of clinicians, clients, or family members or explicating the principles of open dialogue and they reported generally positive experiences. However none of the studies evaluated the acceptability (beyond likeability) or feasibility of implementing the open dialogue approach. Most of the studies conducted by investigators examined modified approaches, informed by the open dialogue principles raising questions as to the practice fidelity of the approach implemented [9].

It is acknowledged that significant time effort and money are required to establish evidence-based practice procedures usually beginning with standardisation of the intervention followed by efforts to demonstrate the feasibility of implementing it and its ability to improve outcomes. It is also clear more rigorous research is needed [8, 9]. This study provides an overview of how Open Dialogue has been operationalised in mainstream UK mental health services and the first evidence of clinical, social and satisfaction outcomes.

### Traditional Services

The study is based in the National Health Service (NHS) mental health trust based in Kent, which is a semi-rural county in South East England where people experiencing a mental health crisis are typically offered a referral to, either the Crisis Resolution and Home Treatment Team (CRHT) or, the Community Mental Health Team (CMHT). The CRHT consists of a multidisciplinary team of nurses, occupational therapists, support workers and psychiatrists providing urgent 24/7 response to people in a mental health crisis. The CMHT is a multidisciplinary team of nurses, occupational therapists, psychologists, support workers and psychiatrists providing routine responses to people with secondary care mental health needs during 9am to 5 pm on weekdays. Service users presenting in crisis are moved to another team, usually CMHT, when the crisis has diminished, although different teams including, for example, the Community Forensic team serve different parts of the service user pathway.

### Peer Supported Open Dialogue (POD) in Kent

Peer Supported Open Dialogue is an adaption of Open Dialogue model specifically designed to be applied for the NHS in the UK [10]. POD adheres both to the organising principles of Open Dialogue [5] (Ziedonis D, Olson M, Seikkula J: 10 Organizational criteria of open dialogue, unpublished) as well as the key elements of dialogic practice whilst also including peer support workers as trained and equally active members of the team. The service development was guided by research and discussions focussing on the value of peer support workers within an Open Dialogue service in terms of similarity in approach to mental distress as well as challenges that may be present [11–13]. Connections and relationship building are central to both Open Dialogue and peer work. There is some evidence that Open Dialogue and peer support share views on human behaviour and alternative explanations for mental health crises, using a shared language building on synergistic understanding of distress [11, 13].

Peer support workers were employed members of the clinical team providing a unique contribution to service users utilising their lived experience as part of the clinical team. Peer support workers have an insight into the difficulties of using mental health services and by sharing their own lived experiences they can build trust and engage people in their treatment [14]. This role also increases social inclusion for service users, helps stabilise subsequent employment or education [15] and improves clinical outcomes with less inpatient bed use [16]. It has also been proposed peer support workers may be valuable in situations where people have little or no social support. Support from peers who have lived experience walk alongside individuals and families through use of mutuality and reciprocity, supporting them in recovery [17]. In Kent, we introduced a standalone team working with the Peer Supported Open Dialogue model [18] to enable more psychological continuity, in keeping with the organisational principles of Open Dialogue [14]. This ensured the POD team would not be passing service users from POD trained practitioners in a CRHT to POD trained practitioners in a CMHT. The team, and thus the same workers, would be with the service user through the crisis and into recovery negating the need for a transition between teams. We discounted integrating POD into an existing CMHT as this may have limited adherence to the model. The POD team was very small at the outset, six whole time equivalent staff which has since has grown to nine whole time equivalent staff.

### Training

All POD staff underwent a one-year training course provided by the Academy of Peer Supported Open Dialogue (APOD) [19]. Members of the POD team, including peer workers had either completed a one year diploma in Open Dialogue practice or were in the process of completing their training. One person, the team manager who also delivered clinical care, undertook the post graduate qualification in Dialogical Approaches in Couple and Family Therapy, psychotherapy trainers training (supervisor level training) accredited by the University of Jyvaskyla, Finland.

### Criteria

We agreed the criteria for the team to accept those presenting in crisis, in a new episode of care, whether via the Single Point of Access or sent by the Crisis Resolution and Home Treatment Team. These teams determined eligibility to NHS secondary specialised mental health care. Due to the small size of the team, it accepted five referrals per week. Care was delivered through network meetings which were attended by staff including peers with two staff usually allocated to attend each network meeting.

### Practice Adherence

We used the Dialogic Practice Adherence tool to reflect on network meetings. It was a helpful tool not only to reflect on adherence but also to help the team develop their relationships and dialogue with each other. We used the tool after every POD network meeting. We were informed in our service design by the unpublished organisational criteria of open dialogue as developed by Zeidonas and Olsen. Both tools are supplementatry documents to this paper.

### Network meetings

Social networks meetings are formed from first contact when a referral is received in order for the person of concern and their identified family members/friends to be present at the first and ongoing meetings. Thus, networks are formed (with professionals also present) in a non-hierarchical way so that all present are able to contribute to the understanding of the crisis**. **Service users had the opportunity to ask others to attend. If only the service user attended the meeting, in some cases, they were encouraged to bring into sessions the inner voices of their family or friends and would be asked “what would ***** say if they were present?”.

The service user and their network identified the topics and agenda items to be discussed in the meeting ensuring that what is important to them and their wellbeing is privileged above other possible professionally identified issues. A relational way of thinking and asking questions was used to bring greater clarity to the situation and provide more opportunities to co-create the meaning of the presenting issue.

The social network set the primary agenda for the meeting. It became apparent during the course of delivery that the reflective process within the meeting became increasingly important in facilitating curiosity and a greater understanding of what had happened and was happening. This process informed the collaborative decision-making at the end of the network meeting. Planned network meetings were based on need, as perceived by the service user and their network, and determined at the end of each network meeting.

### Supervision

The approach required a reflective space for clinicians and the peer support workers to have time to consider their practice and relational way of being with each other and the service users and social network [20–22].

### Location

Our team was located in Kent in a University City. This ensured the service users seen were a mix of those living in the small city as well as the countryside. The team functioned alongside the existing mental health services which include the traditional teams entry points for inpatients; crisis resolution and home treatment, community mental health team, early intervention in psychosis and specialist teams.

### Aims

The overall aim of this study was to implement a POD service in a mainstream NHS setting and examine the changes in a range of user outcomes over a period of six months. These were achieved through our two objectives:

Objectives:

1. To examine service user clinical outcomes of wellbeing and experience during the course of a POD intervention at three time points (pre-POD, three months following POD and six months following POD). To record the number of service users receiving POD who were in employment or full-time education
2. To examine the wellbeing of the family and social network receiving treatment in the POD service at three time points (pre-POD, three months following POD and six months following POD).

## Methods

### Design

This was an exploratory study using a before-after design with participants recruited to the study followed up for six months following on from their first POD meeting.

### Participants

The study was conducted in one locality; a University City in Kent, South East England. Three participant groups were recruited: service users, family and/or social network members, and POD practitioners. Service users were eligible if they were aged between 18 and 70, were experiencing a mental health crisis and would have normally been seen by a traditional mental health service; in this case the Crisis Resolution Home Treatment team (CRHT). People with a learning difficulty or dementia or first episode psychosis were seen by other services and excluded from the study. A convenience sampling approach was used with all service users meeting the eligibility criteria and who had capacity to consent to participate being invited to participate in the study.

Service users coming in to the service were assessed regarding capacity to consent and ability to take part by the clinical team, and information sheets left with them prior to informed consent being obtained. This approach continued until a target of 50 participants was reached. We also recruited any member of their family or social network who consented to the study. The choice to cut off recruitment at 50 participants was guided by the central limit theorem with the sampling distribution of the mean of any independent random variable considered normal if the sample size is large enough. Kirkwood and Sterne suggest in most circumstances, a sample size of 15 and more is enough to give a close approximation to normality [23] with many researcher proposing variables in samples of *n* > 30 can be deemed as normally distributed [24, 25]. We chose a larger figure of 50 which allowed us to undertake data analysis under the assumption that the data are normally distributed.

A flow diagram of the study participants is included as a supplementary document*.*

### Intervention

Service users accessed the POD service in the same way as other local mental health services Individuals who met the eligibility criteria for secondary/specialist mental health care, were in mental health crisis and needed an urgent response, were referred to the POD team who followed the principles of Open Dialogue practice noted above. The multidisciplinary POD team comprised of clinicians from a variety of professional backgrounds and included peer support workers, including a carer, who had trained in POD [10]. The team provided both urgent and routine responses. During the initial stage of a mental health crisis, meetings could take place every day for the first ten to twelve days and could last for up to two hours to foster an adequate sense of security. Subsequent meetings were organised less frequently according to a joint plan agreed by all parties. Similarly, a point of discharge was a shared decision between service user, social network and clinicians.

### Procedure

Following on from an initial referral, allocation for initial contact to POD was made by single point of access, a POD practitioner from the team contacted the service user to ask if the POD team could meet with them at a convenient time and location. They were also asked who else they would like to attend. The initial POD meeting consisting of the clinical and social network participants was held within 24 h of first contact with the POD team. The duration and frequency of POD meetings were not fixed and were decided within the meetings using a case-by-case approach. Where POD was not offered, people in acute mental health crisis would be assessed and supported by CRHT on a daily basis by clinicians working on a shift basis through 24/7. When the crisis abated and in line with CMHT inclusion criteria the person would be followed up by the CMHT, operating weekdays (9am-5 pm) whereby a health care professional supports the person and determines the appropriate clinical intervention.

### Measures

Demographic information was recorded from the case notes. Table 1 outlines the measures used to assess service user’s clinical outcomes, wellbeing, and impact on daily routine, family/social network support, as well as the assessment time points. Measures completed by service users were compiled into questionnaire booklets and were either completed with the help of an independent researcher or completed independently by the service user. The number and frequency of contacts with the POD team were collected centrally.

**Table 1:** Outcomes Measures

Name	Measuring	Description	Measured at
Health of the Nation Outcome Scales (HoNOS) [26]	Health and social functioning of people with severe mental illness	12 items measured on 5 point scale; Range 0–48; Higher scores equate to lower functioning (clinician rated)	Baseline and Discharge from service
Shortened Warwick-Edinburgh Mental Well-being Scale (SWEMWBS) [27]	Mental Wellbeing	7-item measure; 5-point scale; Range 7–35; High mental wellbeing 28 or above; (self-reported by service user)	Baseline, 3 and 6 months
Work and Social Adjustment Scale (WASAS) [28]	Impairment in functioning	5-item measure; 9-point scale; Range 7–35; Higher scores equate to greater impairment (self-reported by service user)	Baseline, 3 and 6 months
NHS Community Mental Health Survey (CMHS) [29]	Experiences of health and social care received through NHS mental health services	42 questions. Mean score recorded (from 1–10). Higher score equates to more positive experiences (self-reported by service user)	Baseline, 3 and 6 months
Carers Wellbeing and Support Scale (CWS) [30, 31]	Carers satisfaction of support received	17-item ‘support’ sub-measure; 4-point scale. Range 0–51. Higher scores equate to perceived greater levels of support (self-reported by family/carer)	Baseline, 3 and 6 months

### Statistical Analysis

IBM SPSS Statistics v27 software was used for the statistical analysis using anonymised data. Descriptive information was recorded for all the measures. For the HoNOS, SWEMBS, WASAS, CMHS and CWS measures, a one way ANOVA with repeated measures was carried out to examine the three group means (two group means for HoNOS) across the three time points with post-hoc Bonferroni corrections used to determine the significance level for differences between the individual time points. Mauchly's Test of Sphericity was used to ensure the assumption of sphericity had not been violated. The distribution of the dependent variable in the three related time-point groups (two in the case of HoNOS) was measured by the Shapiro–Wilk test of normality where *p* =  < 0.05 indicated the data significantly deviated from the normal distribution.

The Friedman test is the non-parametric alternative to the one way ANOVA with repeated measures and was used to test for differences between groups on any continuous data dependent variable that significantly deviated from the normal distribution by scoring *p* =  < 0.05 on the Shapiro–Wilk test of normality. Post hoc analysis with Wilcoxon signed-rank tests was conducted, with a Bonferroni correction applied, to determine the significance level for differences between the individual time points. The Friedman test was also used when *n* =  < 30 as we could not assume data from samples of less than 30 were normally distributed.

Descriptive details are reported for the mean scores for hospitalisation rates and contact rates.

## Results

During the study period 113 service users were approached to participate in the study until the required 50 participants were recruited; a recruitment rate of 44.2%. Of those further 63 service users, 18 (15.9%) declined to take part, 4 (3.5%) were unable to give informed consent, 24 (21.2%) were either discharged or disengaged with the service before they could be recruited, and the remaining 17 (15%) did not take part for unknown reasons. In addition, 25 carers consented to take part in the research although four carers did not complete any outcome measures. There were slightly more male participants (27—54%) with the overwhelming majority (47 – 94%) being white British. The mean age was 35.8 years.

### Attrition

Every participant completed the baseline measures; 40 (80%) completed the 3 month measures and thirty-seven (74%) completed the 6 month measures. The main reasons for not completing the measures were either that the service user had disengaged from the service or they had been discharged from the caseload.

### Network meeting attendance

We examined the attendance at the first ten network meetings for all participants, or for all meetings with those participants discharged before they completed ten network meetings. This resulted in records of attendance for 467 meetings. Just over half the meetings (245—52.5%) were attended by the service user and other network members while the remaining meetings (222 – 47.5%) were solely with the service user. In those meetings where network members attended, the most common attendee was the spouse or partner of the service user (97 instances; occurring in 39.6% of the network meetings). This was followed by one parent (74—30.2%), both parents (31—12.7%), friends (30—12.2%), adult children or other relatives (24—9.8%), siblings (20—8.2%) and others (5—2%).

#### Clinical symptoms, experience of service, wellbeing, impact on daily routine and carer support

Table 2 presents the mean scores of the outcome measures from three user self-report measures and one carer/family member self report measure at three time points. One clinician-rated measure is also reported at baseline and the six months time-point. All five measures show improvements from the baselines score at the 3 and 6 month time point. The table reports the results of the Shapiro Wilks test for normality.

**Table 2:** Clinical Outcomes, Wellbeing, Clinical Experience, Impact on Daily Routine and Carer Support Scores

Measure	Baseline		Three Months		Six months	
	*n* =	Mean (sd) W test statistic (df), and *p* value	*n* =	Mean (sd) W test statistic (df), and *p* value	*n* =	Mean (sd) W test statistic (df), and *p* value
**HoNOS**	50	21.26 (6.65) W(50) = 0.97, *p* = 0.18	n/a	n/a	42	12.31 (7.26) W(42) = 0.20, *p* = 0.143
**SWEMWBS**	49	16.84 (4.44) W(44) = 0.98, *p* = 0.64	40	20.71 (4.38) W(38) = 0.97, *p* = 0.32	37	20.63 (4.53) W(36) = 0.96, *p* = 0.15
**WASAS**	50	24.94 (9.41) W(44) = 0.96, *p* = 0.144	38	18.66 (10.78) W(38) = 0.95, *p* = 0.09	36	16.69 (11.35) W(36) = 0.98, *p* = 0.09
**CMHS**	45	8.44 (1.88) W(44) = 0.76, *p* = < 0.01^a^	40	8.93 (1.86) W(38) = 0.60, *p* = < 0.01^a^	37	9.19 (0.97) W(36) = 0.77, *p* = < 0.01^a^
**CWS** **Support**	21^b^	41.67 (1.88)	16^b^	46.69 (5.85)	13^b^	48.00 (3.85)

^a^ *p* =  < 0.05 indicating the data significantly deviated from the normal distribution

^b^ *n* =  < 30 so cannot assume data were normally distributed

The SWEMWBS scores show an improvement in mental wellbeing between baseline and the 3 month timepoint. The mean score at 6 months is slightly less than the 3 month score but more than at baseline. The WASAS scores record ongoing improvement in self-reported functioning in work and social activities at each time point. The HoNOS scores indicate there was a reduction in clinician-reported symptom severity measured by HoNOS scores between baseline and 6 months/discharge time point.

The CMHS scores indicate an improvement in the care experiences the service users receive with an increase in scores at each time point. The scores at all three time points compared favourably with the overall Trust and national scores for satisfaction with the 6 month POD service recording a mean of score 9.19; compared to the 2017 Trust score of 6.51; and the 2017 national score of 7.03. The CWS Support sub-scale scores record that an increase in support received from services was reported between baseline and 3 months and this support score increased at the 6 months’ time point.

The tests for normality showed the HoNOS, SWEMBS and WASAS data were normally distributed. The test for the CMHS data concluded that this is probably not normally distributed and the low CWS sample size means we cannot assume the data is normally distributed. Following on from this, the HoNOS, SWEMBS and WASAS data were analysed by a one way ANOVA with repeated measures, and the CMHS and CWS by the Friedman test. The results are shown in Table 3.

**Table 3:** Differences between Clinical Outcomes, Wellbeing, Clinical Experience, Impact on Daily Routine and Carer Support Scores at Different Time-Points

**Measure** (n)	**Baseline** Mean (sd)^a^/ Median (iqs)^b^	**Three months** Mean (sd)^a^/ Median (iqs)^b^	**Six months** Mean (sd)^a^/ Median (iqs)^b^	**F (df)**^**a**^**/** **χ**^**2**^** (df)**^**b**^	***p* value**
**HoNOS** (*n* = 42)	20.95 (6.4)^a^	n/a	12.31 (7.26)^a^	62.45 (1)	< 0.01
**SWEMWBS** (*n* = 33)	16.19 (3.89)^a^	20.45 (4.48)^a^	20.67 (4.68)^a^	24.05 (2)	< 0.01
**WASAS** (*n* = 32)	27.28 (9.36)^a^	19.75 (11.17)^a^	18.81 (11.66)^a^	18.63 (2)	< 0.01
**CMHS** (*n* = 31)	9 (7–10)^b^	10 (8–10) ^b^	10 (9–10)^b^	11.02 (2)^b^	< 0.01
**CWS** (*n* = 10)	45.5 (42–47.5)^b^	47 (43.75–50.25)^b^	49.5 (47.25–51)^b^	12.86 (2)^b^	< 0.01

For the analysis, the independent variable needed to consist of at least two categorical "related groups”. This resulted in participant data only being included if it had been collected at all three time-points (or both time-points for the HoNOS data).

Mauchly’s Test of Sphericity was performed on the SWEMWBS and WASAS with *p* =  > 0.05 being recorded for both sets of data indicating the assumption of sphericity had not been violated and that the one way ANOVA with repeated measures test was appropriate. The table records the mean scores, standard deviation at all three time points with the one way ANOVA with repeated measures scores for HoNOS, SWEMWBS and WASAS. The median scores, with the inter quartile range, as well as the Friedman test scores are recorded for CMHS and CWS.

The analysis revealed that there were significant differences between the scores at different time points for all five measures. All reported *p* =  < 0.01. The HoNOS scores were significantly better at six months when compared with the baseline scores. Post hoc analysis, with the Bonferroni correction applied, was undertaken on the other four sets of data to ascertain the significant differences between different time points. Statistically significant improvements were found with the SWEMWBS scores reported at three months and six months compared with the baseline well-being scores. There was also a small improvement in well-being scores reported at six months when compared with the three months score but this was not significant. Similarly, the WASAS scores recorded significant improvements in functioning at the three month and six month time points when compared with the baseline scores. A small non-significant improvement in the six month scores was reported compared to the three month score. There were significant improvements at six months when compared with baseline scores in the experiences of health and social care as reported in the CMHS. There were also improvements reported at the three month and six month time-points in the support received by carers/families as recorded in the CWS support sub-scale with a significant improvement found between the baseline and three month scores.

#### Employment or Education

At baseline 22% were in full-time employment (*n* = 11) and 12% were in full-time education (*n* = 6). At the six month point 30% were in full-time employment (*n* = 15) and 18% were in full-time education (*n* = 9), indicating a 14% increase in take up of employment or education.

#### Observations on People using POD services

Observations on 171 POD service users are presented to give some indication of service use during the period of the study. It is acknowledged that a number of external factors may have influenced these observations and should be treated with caution. The outcomes are recorded as raw scores with no inferential analysis undertaken.

### Hospitalisation rates

The mean number of bed days for the service users receiving the POD service was 0.44 (sd 2.8) bed days in hospital during their complete period of continuous care starting from their first referral with to date of their final discharge.

### Contact rates

The mean duration of contact between the POD service and users of the service was 88.14 (sd 89.91).

## Discussion

This is the first study to evaluate the POD approach in a large NHS mental health trust, delivering a standalone POD team, as complete care to NHS mental health service users presenting in crisis within a community setting. This model was set up from scratch to test it in the NHS and this has proved possible. There were a number of barriers to this POD team model as, contrary to most NHS service models, it worked trans-diagnostically and across crisis and recovery discrete community pathways of traditional services. The POD team also in-reached to inpatient services.

We collected data from service users, network members and clinicians regarding the implementation and performance of the POD approach. Aspects investigated included; self-reported and clinician-reported clinical outcomes, satisfaction of service users and network members; and recorded broad data for the same time period between community and crisis teams. The group of service users treated were predominantly young people, mean age 35 years, slightly more males than females in an area where generally the population is white British, with a student population attracted by the local universities. The approach is organised for a shared decision making process. In health services there is an emerging focus on co-production and active citizenship in recovery [32] such that there is a strong case for enhancing the implementation of shared decision making to promote recovery. This is fundamental to POD which is democratic, collaborative and sees each person in the network meeting as an equal or potential partner in recovery. In a service user led report it was identified that most service users who took part in the project which gathered their views felt that social approaches to mental health, which take account of the whole person and wider societal issues affecting them, are the most helpful [33].

### Clinical Outcomes

POD clinical outcomes are encouraging with all clinical measures of service user outcomes showing benefits in terms of recovery and function from three self-report measures and one clinician-rated measure. All four measures show improvements from the baselines score at the 3 and 6 month time point with statistically significant improvements recorded in all the outcomes. There are continual improvements for all measures from baseline to 3 months and also from 3 to 6 months. The SWEMWBS scores show there was a significant improvement in mental wellbeing between baseline and the 3 month timepoint. The significance of the 4.48 reduction in the SWEMBS score between baseline and 6 months can be viewed in that a change of between 1 and 3 points is reported to meet the thresholds for statistically important change [34]. The WASAS scores record ongoing improvement in self-reported functioning in work and social activities at each time point with the scores between baseline and 3 months being significantly different. The WASAS score at baseline was 27.28 with a score above 20 suggesting moderately severe or worse psychopathology. This contrasts with WASAS scores of between 10 and 20 (as recorded by this cohort at 3 months and 6 months) which are associated with significant functional impairment but less severe clinical symptomatology [35]. This suggests that improvements in clinical outcomes happen reasonably quickly but are maintained with some additional improvements after the 3 month period. These results show benefits of reduced symptom severity, improved mental wellbeing and increased engagement in work and social activities. These improvements go hand in hand with an improved quality of life for the service users and indicates reduced distress and care needs with a better prognosis for their future. This is of great significance to service users.

There are statistically significant improvements for CMHS between baseline and 6 months. For satisfaction, it appears the satisfaction score is already high at baseline but improves, and is maintained whilst they are receiving POD. This approach appears to be well accepted by service users and a positive experience.

National Health Service user experiences are measured each year using the CQC Community Mental Health Survey. Our observations of the scores show that service users rated the POD service more highly than other local and national data and suggests this approach may address some outstanding issues with mental health services around service user satisfaction.

Evaluation is the basis for improving care and it has been suggested that mental health care services can be evaluated on two dimensions; whether they are beneficial or harmful, and whether they offer value for money [36]. This service was demonstrated to be beneficial to service users in terms of clinical outcomes and overall satisfaction. It incorporates shared decision making and the option to mobilise social support networks with a high level of choice offered to the service user and this may address national quality issues of service user satisfaction identified by the Care Quality Commission [37].

Clinical outcomes were consistent across mental wellbeing and work/social adjustment (self-reported), CMHS and HoNOS scores (clinician-reported) and showed that this approach is clinically effective with significant improvements across all measures. The Healthy London Partnership guide to analysing HoNOS data suggest that using effect size statistics such a Cohen’s d can aid interpretation of HoNOS data because it calculates the significance of the change. The change between the mean score recorded at 6 months as opposed to baseline equates to a Cohen’s d effect size of 1.24. It is proposed that any effect size of over 0.8 is indicative of an improvement of critical clinical importance [38]. Reduced symptoms, better functioning with service satisfaction are highly desirable goals of any mental health service and even small improvements can have a clinically valuable benefit for those in receipt of and providing services in terms day to day life and work fulfilment.

### Hospitalisation rates

The mean number of bed days for the service users receiving the POD service was 0.44 in hospital during their complete period of continuous care starting from their first referral to date of their final discharge. This seems a low figure and, as a reference point, traditional services reported a mean number of 2.24 bed days for service users accessing the locality CRHT/CMHT services over the same period; over five times higher. The general observations of POD relative to traditional community care are of interest and point to the need for further work looking at comparisons. In general it is notable that any reduction in bedday usage is highly impactful on cost of care within the health system as a whole and needs further exploration and research. A long term data analysis will be needed before conclusions can be drawn about cost-effectiveness and value for money.

### Contact rates

The mean duration of each contact between POD services and users was 88.14. This level of contact can be seen in context when looking at the reported average duration of 62 min for meetings between CRHT/CMHT traditional services and service users. This is congruent with the POD model which is responsive to need as perceived and expressed by the service user rather than on the basis of appointment slots or capacity of clinicians in clinics unless a service user declares their issue urgent in traditional services when they then receive duty worker support. The decision about frequency of contact is made by the service user with the team and this may have been empowering for the service user, a collaborative approach which may have improved the outcomes for service users. This could be explored in greater detail in future studies. The current evidence tentatively indicates improved outcomes when family and social network are involved in terms of reduced hospitalisation and recovery [39, 40].

### Wellbeing of family and social network

The CWS support scale scores seem favourable given the findings of Quirk et al. [31] during the development of the measure. However the population under investigation in the Quirk study were different so the findings are not directly comparable. The 6 month score of 48.0 in our study shows a marked increase in the how carers perceived they were being supported by the POD team. It is also striking to note that at baseline the POD participants record a score of 41.67 suggesting that carers acknowledged the initial support provided by the POD service even at this early stage of the intervention.

There seems to be a wish for the involvement of family and friends as over half the meetings were attended by carers. When carers are involved in care, they are more involved in the service users recovery and with reduced risk of mortality by suicide [39]. One of the strengths of the POD model lies in the mobilisation of the social network. In this study it was found that almost half of the meetings did not have any network members present. Possible reasons for this could be due to stigma or a low level of social support in this population overall. Our data is for network attendance at the first ten meetings and it would be useful in future studies to monitor this data for all network meetings and observe any variations. However having carers involved in the half the total meeting with service users is much higher than our own clinical experiences of community services general involvement of family and support networks. Typically service users are seen singly and then family and social network consulted at the conclusion of the appointment if at all [40–42]. The assessment of service users alone often leads to concern from families about not being consulted. We acknowledge though that an evaluation of how often family and social network are involved in ordinary consultations needs to be considered objectively. Despite this finding, the high levels of satisfaction suggest that the POD team provided suitable support for individuals when other support was absent or unwanted. This could be explained by the POD team’s practice of engaging with important voices through the service user as a way of understanding relational aspects of the service user. The meetings contained the same dialogical conversations whether the service user was alone or not with the same decision making process. All the voices were valued in the meetings to emphasise a relational understanding of the service user’s distress. Barriers to shared decision making in practice have been described and can be addressed by shared care planning [40]. POD provides specific training for collaborative working which is likely to enhance the wellbeing in the social network.

### Implementing the Open Dialogue Approach

A review of the evidence for Open Dialogue found that there has been considerable variation in how the Finnish OD approach has been implemented in different locations making comparisons unreliable [8]. The clinicians reflections on the Dialogic Practice adherence tool guided the POD service in treatment practice principles outlined by the Finnish researchers and developers of the Dialogic Practice adherence tool. [1, 5] (Ziedonis D, Olson M, Seikkula J: 10 Organizational criteria of open dialogue, unpublished). High practice adherence is important as there are a number of attempts to set up OD treatments around the world which struggle to be faithful to the practice and fully informed by the organisational criteria. The lack of a validated practice adherence instrument is an impediment to this. This study has looked at the implementation of a true OD practice approach modified only by the addition of peer workers to the clinical team. Ensuring onsistency of the approach being maintained ensures psychological continuity for service users. The Dialogic Practice Adherence tool supported reflection on practice to the approach and helped the team develop in their relationships and dialogue with each other.

Open Dialogue requires a reflective space for clinicians to have time to consider their practice and relational way of being with each other and the service users and social network [15–17]. This enabled a deeper understanding of each other as practitioners but also helped to take account of power positions in meetings including between practitioners. This usually took place without an external facilitator [16]. Essentially this was a model of reflective peer supervision for all members of the team. It is important to note that no clinical decisions were made in this supervision space as those decisions remained in the domain of the social network meetings.

A qualitative study examining the introduction of a POD service in England reported that clinicians positively viewed this way of working [43]. There were, however, mixed views from service users, including being unsure as to the purpose of the network meetings and finding the reflective conversations strange, though, the majority felt listened to and understood. The study was carried out during the training of the clinicians so may not accurately reflect the opinions of an established POD service from users and professionals.

The evidence base for the Open Dialogue approach, in a UK setting, remains sparse and currently there has not been any published quantitative data examining the POD model [43, 44].

### Challenges

This study begins to grapple with a number of challenges in the delivery of Open Dialogue in mainstream NHS services. We were required to negotiate these challenges within the NHS Trust and recruitment to the research was successful having an acceptable level of attrition, with 74% of recruited participants completing the six month measures, allowing quantitative data to be collected.

The team functioned alongside the existing mental health services which included traditional community teams’ entry points for; inpatients, crisis resolution and home treatment, community mental health team, early intervention in psychosis and specialist teams. There were major challenges to develop a service that did not follow the established pathways for entry into care under traditional team boundaries. There were further challenges in continuing to work with people until they felt they had achieved recovery and were ready for discharge rather than the clinician and service design making those decisions as is traditional in the medical model.

We had to remain within the governance structures of a large NHS trust. The standard operating procedure written for the team included agreements with all parts of the system as to how the POD team would interface and work safely within and seamlessly with the usual system of mental health care delivery. For example, we had to consider the Care Programme Approach (care and treatment plan) [45, 46] and risk assessment and management [47] whilst continuing to work dialogically. It was essential not to increase risk to any service user or family member or indeed staff member during the course of this innovation. We also monitored quality and performance in line with all other Trust services.

The Kent POD Team was a small scale clinical approach in the very big system of a mental health trust within the NHS, with approximately 3300 staff serving a population of 1.7 million people. It was also an enormous challenge to continue working with people until they felt they had achieved recovery and were ready for discharge rather than the clinician making those decisions as is traditional in the medical model. Organisational investment is also needed to allow the implementation of service user choices including training clinicians in shared decision making as within the POD model.

#### Strengths

This trial was run in a real life NHS clinical setting taking a pragmatic approach.

#### Limitations

There was also a possibility of bias, in particular social desirability bias, impacting on the findings through the use of self-report measures. The authors aimed to minimise the likelihood of bias through adopting methods to reduce the prospect of social desirability bias detailed by Nederhof [48]. This entailed the team collecting the data from a range of different sources (service users, carers and clinicians), using well established validated measurement tools ensuring the participants and assuring the participants their answers would remain confidential. In addition, any missing data was not included in the analysis. This is also a potential source of bias as some data from the non-respondents could differ systematically from those that responded. This is a small scale study, not randomised and looked at one team. A full randomised control trial is therefore indicated.

## Conclusions

As Peer Supported Open Dialogue Services emerge in the UK, this study provides the first evidence of clinical, social and satisfaction outcomes in mainstream mental health services. A flexible, social network response to crisis care that includes peer support workers as a key component of the care, POD, with the continuity of the same POD clinicians building a shared memory of a family’s distress throughout care, represents a significantly different approach to mental health care in the UK. Clinical outcomes were consistent across mental wellbeing and work/social adjustment (self-reported) and HoNOS scores (clinician-reported) and showed that this approach is clinically effective with significant improvements across all measures. As such, this study evidencing clinical outcomes and satisfaction for service users and their families supports the need for a full scale randomised control trial research at a national level and augurs well for its findings. Since the completion of this study this POD service has gone on to be a site in the Open Dialogue: Development and Evaluation of a Social Network Intervention for Severe Mental Illness (ODDESSI) – UK national research trial. Despite the challenges of introducing a new service that was different to existing NHS service structure and principles, the adapted POD approach allowed that continuity of care from crisis through recovery be prioritised. Peer Supported Open Dialogue is an innovative and clinically effective method of treatment delivery in a large NHS mental health trust.

## Supplementary Information

**Additional file 1.** 

**Additional file 2.** 

**Additional file 3.** 

## Data Availability

The datasets used and/or analysed during the current study are available from the corresponding author on reasonable request.

## Abbreviations

CMHS: NHS Community Mental Health Survey
CQC: Care Quality Commission (NHS UK)
CRHT: Crisis Resolution Home Treatment teams
CWS: Carer Wellbeing and Support Scale
FYFV: The Five Year Forward View for Mental Health
HoNOS: Health of the Nation Outcome Scales
KMPT: Kent and Medway NHS and Social Care Partnership Trust
NHS: National Health Service (UK)
OD: Open Dialogue
POD: Peer Supported Open Dialogue
SWEMWBS: Short Warwick-Edinburgh Mental Wellbeing Scale
WASAS: Work and Social Adjustment Scale
