# Supplementary material for: Peer supported Open Dialogue in the National Health Service: implementing and evaluating a new approach to Mental Health Care
Source: BMC Psychiatry. 2022 Feb 22;22:138. doi: 10.1186/s12888-022-03731-7 (PMC8862567; doi:10.1186/s12888-022-03731-7)
Supplement: Supplementary file 1 — Additional file 1. [file 12888_2022_3731_MOESM1_ESM.pdf]

University of Massachusetts UMASS Medical School

Ziedonis D., Olson M., &Seikkula, J.*

Draft only – do not cite, quote, or distribute without authors' permission

10 Organizational Criteria of Open Dialogue

Agency: ________________ Specific Program (s) in Agency: ______________________________________

* Please use a separate sheet for each program and a separate one for the agency as a whole.

**1. Person and Family (Support Network) Centered Care Approach?**	**___Yes ___No ___Partial**
**2. Culture which values & demonstrates respect, authenticity & collaboration?**	**___Yes ___No ___Partial**
**3. Clinical teams (with multiple providers) meet routinely with person and family (support network) in collaborative meetings (even if limited to primarily when making major decisions)?**	**___Yes ___No ___Partial**
**4. Staff is well trained in respectful communication, dialogic practice and family/network engagement?**	**___Yes ___No ___Partial**
**5. Create a welcoming environment with a focus on customer experience?**	**___Yes ___No ___Partial**
**6. Provide and connect services in clinical and community settings?**	**___Yes ___No ___Partial**
**7. Practice the 12 Key Elements of Dialogic Practice including tolerating uncertainty and promoting dialogue?**	**___Yes ___No ___Partial**
**8. Provide immediate support and access to needed services?**	**___Yes ___No ___Partial**
**9. Shared decision-making process?**	**___Yes ___No ___Partial**
**10. Use Open Dialogue as a mindful way of being?** In clinical work:<br>In non-clinical team work:	**___Yes ___No ___Partial**<br>**___Yes ___No ___Partial**

© 2015 University of Massachusetts Medical School v2. 6/19/15

***The authors share the copyright of this work. The material may be distributed in whole with the authors' permission. Please contact Dr. Ziedonis for permission at Douglas.Ziedonis@umassmemorial.org**
