# Supplementary material for: Peer supported Open Dialogue in the National Health Service: implementing and evaluating a new approach to Mental Health Care
Source: BMC Psychiatry. 2022 Feb 22;22:138. doi: 10.1186/s12888-022-03731-7 (PMC8862567; doi:10.1186/s12888-022-03731-7)
Supplement: Supplementary file 2 — Additional file 2. [file 12888_2022_3731_MOESM2_ESM.pdf]

University of Massachusetts
UMASS Medical School

Subject #: __________

Date: ___/___/___

Session #: __________

Key Elements of Dialogic Practice in Open Dialogue: Fidelity Chapter

Dialogic Practice Adherence Scales

Olson, M., Seikkula, J., & Ziedonis, D.
Draft only—do not cite, quote, or distribute without authors' permission

The Twelve Key Elements of Fidelity to Dialogic Practice in Open Dialogue:

I. Two (or More) Therapists in the Team Meeting: Yes / No

II. Participation of Family and Network
Is anyone from the identified person's network in the room? Yes / No
   a. If yes, what is his/her relation to the patient?
      1. ________________
      2. ________________
      3. ________________

III. USE OF THREE SUBCATEGORIES OF OPEN-ENDED QUESTIONS: To what extent did the clinician open the treatment meeting with open-ended questions to build the structure of the dialogue?
   a. Introduction: Were the following 2 questions asked? (skip this question if not a 1st meeting):
      1. What is the history of the idea for the meeting? Yes / No
      2. How does each person (ID person / Network members) want to use the meeting? Yes / No
   b. Subsequent Appointments:
      1. Use of 2nd open-ended question to begin session? Yes / No

   c. Ongoing use of open-ended inquiry questions throughout the treatment meeting as a way of linking client utterances and building the dialogue. To what extent did the clinician use open-ended questions to engage everyone to build the structure for subsequent dialogues?

Extensiveness/Adherence Rating (Open Ended Questions):

1--------------------2--------------------3--------------------4
Not at all | Somewhat | Considerably | Extensively

Skill/Competence Rating (Engaging with the individual / network):

1--------------------2--------------------3--------------------4
Unsatisfactory | Fair | Good | Excellent

© 2014 University of Massachusetts Medical School

IV. RESPONDING TO CLIENT "UTTERANCES" (verbal and non-verbal communication):

a. *RESPONSIVE LISTENING TO MAKE SPACE FOR STORIES THAT ARE NOT YET TOLD:* To what extent was the clinician responsive to the client's talk and gestures in a way that invites a further response? For instance, responsiveness can be evident when there is a change during the meeting in the direction of a calmer atmosphere and a conversation that allows for pauses and more joint reflection about issues in a dialogical way.

Extensiveness/Adherence Rating:

1--------------------2--------------------3--------------------4
Not at all Somewhat Considerably Extensively

Skill/Competence Rating:

1--------------------2--------------------3--------------------4
Unsatisfactory Fair Good Excellent

b. *USE OF CLIENT'S WORDS:* To what extent did the clinician actively follow what the client says and integrate the client's very same words and phrases into his/her responses?

Extensiveness/Adherence Rating:

1--------------------2--------------------3--------------------4
Not at all Somewhat Considerably Extensively

Skill/Competence Rating:

1--------------------2--------------------3--------------------4
Unsatisfactory Fair Good Excellent

c. NONVERBAL ATTUNEMENT. To what extent did the therapist show attunement and synchronize their responses to client's gestures and the volume and variation in their tone of voice.

Extensiveness/Adherence Rating:

DRAFT

1-------------------2-------------------3-------------------4
Not at all Somewhat Considerably Extensively

Skill/Competence Rating:

1-------------------2-------------------3-------------------4
Unsatisfactory Fair Good Excellent

d. ALLOWING FOR AND TOLERATING SILENCES IN THE CONVERSATION:

Extensiveness/Adherence Rating:

1-------------------2-------------------3-------------------4
Not at all Somewhat Considerably Extensively

Skill/Competence Rating:

1-------------------2-------------------3-------------------4
Unsatisfactory Fair Good Excellent

V. EMPHASIZING THE PRESENT MOMENT:

a. IMMEDIATE REACTIONS: To what extent did the clinician respond and even shift the dialogue to address immediate reactions that arise in the conversation, emphasizing the present moment of the meetings, while listening to the stories of what has happened in client's life?

b. WHEN EMOTIONS NATURALLY AROSE: To what extent did the therapist make space for and stay present with emotions (including emotions of members of the family/network) in a safe way, but not to interpret their emotional, embodied reactions?

Extensiveness/Adherence Rating:

1-------------------2-------------------3-------------------4
Not at all Somewhat Considerably Extensively

Skill/Competence Rating:

1-------------------2-------------------3-------------------4
Unsatisfactory Fair Good Excellent

DRAFT

VI. ELICITING MULTIPLE VIEWPOINTS: POLYPHONY

a. *OUTER POLYPHONY*: To what extent did the clinician engage everyone in the dialogue, allowing for everyone to be heard in an equal way, and have the possibility of speaking, integrating incongruent language, and managing a dialogue (vs. monologue)?

Extensiveness/Adherence Rating:

1--------------------2--------------------3--------------------4
Not at all Somewhat Considerably Extensively

Skill/Competence Rating:

1--------------------2--------------------3--------------------4
Unsatisfactory Fair Good Excellent

b. *INNER POLYPHONY/VOICES OF THE CLIENT*: To what extent did the clinician invite multiple and possibly conflicting viewpoints or voices? One way of doing this is to engage absent members of the social network as voices in the inner dialogue. Another example is to ask for different point of views of a single person concerning the discussion topic.

Extensiveness/Adherence Rating:

1--------------------2--------------------3--------------------4
Not at all Somewhat Considerably Extensively

Skill/Competence Rating:

1--------------------2--------------------3--------------------4
Unsatisfactory Fair Good Excellent

VII. USE OF A RELATIONAL FOCUS IN THE DIALOGUE: To what extent did the clinician focus on the relational aspects of the spoken stories? This can be emphasized by, for instance, the use of circular questions to address more than one person, to define the relationships in the family, and to express interests in the relational issues?

Extensiveness/Adherence Rating:

1--------------------2--------------------3--------------------4

DRAFT

© 2014 University of Massachusetts Medical School

Not at all Somewhat Considerably Extensively

Skill/Competence Rating:

1-------------------2-------------------3-------------------4
Unsatisfactory Fair Good Excellent

VIII. RESPONDING TO PROBLEM DISCOURSE OR BEHAVIOR AS MEANINGFUL ("NORMALIZING TALK"): To what extent did the clinician strive to comment and respond to what was said in a way that sees symptoms or problem behavior as "natural" responses" to stressful life situations? To what extend did the clinicians listen, with genuine interest and from the point of view of the speaker, to client's descriptions of experiences and behavior that may sound strange.

Extensiveness/Adherence Rating:

1-------------------2-------------------3-------------------4
Not at all Somewhat Considerably Extensively

Skill/Competence Rating:

1-------------------2-------------------3-------------------4
Unsatisfactory Fair Good Excellent

IX. EMPHASIZING CLIENT'S OWN WORDS AND STORIES (I.E. WHAT HAS HAPPENED IN A PERSON'S LIFE) – NOT SYMPTOMS: To what extent did the clinician help the client find words to gain access to language for and communicate more clearly about difficult experiences? This is visible, e.g., in how much therapists take into account those issues that clients utter only in one word or in sub-sentences that may be relevant in the problem situation?

Extensiveness/Adherence Rating:

1-------------------2-------------------3-------------------4
Not at all Somewhat Considerably Extensively

Skill/Competence Rating:

1-------------------2-------------------3-------------------4
Unsatisfactory Fair Good Excellent

DRAFT

X. IN WHAT WAY DID THE PROFESSIONALS TALK WITH EACH OTHER IN THE MEETING WHILE THE FAMILY AND NETWORK WERE PRESENT (the reflecting process, making treatment decisions, and asking for feedback): There are three parts; the first two are interchangeable, but the last one follows the professionals' conversation.

Is there a separate designated reflecting team present (Norway/Tom Andersen "Reflecting Process" variation) or are reflections occurring spontaneously amongst professionals who are already present in the treatment team dialogue? (Finland "Reflecting Talk") – Please select one:

a. "Reflecting Process" (Norway Version) ____
b. "Reflecting Talk" (Finland Version) ____

c. To what extent did the clinicians reflect upon their own ideas/images/associations, with the client and family present?

Extensiveness/Adherence Rating:

1-------------------2-------------------3-------------------4
Not at all Somewhat Considerably Extensively

Skill/Competence Rating:

1-------------------2-------------------3-------------------4
Unsatisfactory Fair Good Excellent

d. To what extent did the clinician create an opportunity for the family to respond to the professionals' discussion on/and interpretations?

Extensiveness/Adherence Rating:

1-------------------2-------------------3-------------------4
Not at all Somewhat Considerably Extensively

Skill/Competence Rating:

1-------------------2-------------------3-------------------4
Unsatisfactory Fair Good Excellent

XI. BEING TRANSPARENT: To what extent did the clinician disclose information on all discussions that took place at the treatment meeting to all members present, allowing each person to be equally privy to information on hospitalization,

DRAFT

medication, treatment alternatives etc.? Transparency is evident both in the reflecting process and in the way the clinician responds to the client.

Extensiveness/Adherence Rating:

1--------------------2--------------------3--------------------4
Not at all Somewhat Considerably Extensively

Skill/Competence Rating:

1--------------------2--------------------3--------------------4
Unsatisfactory Fair Good Excellent

XII. Tolerating Uncertainty:

a. To what extent did the clinician continue to listen to and reflect on client stories, while delaying formalized assessment or rushing to treatment planning?

Extensiveness/Adherence Rating:

1--------------------2--------------------3--------------------4
Not at all Somewhat Considerably Extensively

Skill/Competence Rating:

1-----------------2--------------------3--------------------4
Unsatisfactory Fair Good Excellent

b. How available are the clinicians? Is the team available to meet with the family as often as needed?

Extensiveness/Adherence Rating:

1--------------------2--------------------3--------------------4
Not at all Somewhat Considerably Extensively

Skill/Competence Rating:

1-----------------2--------------------3--------------------4
Unsatisfactory Fair Good Excellent

DRAFT

c. Is the clinician trying to make a connection, understand and respond to a whole person in a context, rather than reacting to isolated behaviors?

Extensiveness/Adherence Rating:

1--------------------2--------------------3--------------------4
Not at all Somewhat Considerably Extensively

Skill/Competence Rating:

1-----------------2--------------------3--------------------4
Unsatisfactory Fair Good Excellent

DRAFT
