# Supplementary material for: Peer supported Open Dialogue in the National Health Service: implementing and evaluating a new approach to Mental Health Care
Source: BMC Psychiatry. 2022 Feb 22;22:138. doi: 10.1186/s12888-022-03731-7 (PMC8862567; doi:10.1186/s12888-022-03731-7)
Supplement: Supplementary file 3 — Additional file 3. [file 12888_2022_3731_MOESM3_ESM.docx]

**Figure: Flow Diagram of Study Participants**

Users of POD service approached

n =113

n = 18 declined to take part

n = 4 unable to give informed consent

n = 24 discharged/disengaged

n = 17 unknown reasons

Baseline assessments

Service users n = 50

Carers n =25

Three month assessment

Service users n = 40

Carers n = 16

Six month assessment

Service users n = 42

Carers n =13
